# Eradication of *Helicobacter pylori*: a prospective comparative randomized trial of standard versus optimized quadruple therapy

**DOI:** 10.2144/fsoa-2023-0316

**Published:** 2024-05-15

**Authors:** Salma Souissi, Cyrine Makni, Basma Chaieb, Amine Jarraya, Nadia Toulgui, Lobna Jmal, Mouna Mlika, Chadlia Fendri, Mohamed Bouchoucha, Rabie Razgallah, Leila Belhadj Ammar, Olfa Bousnina, Fawzi Mezni, Awatef Jmal, Lamia Kallel

**Affiliations:** 1 University of Tunis El Manar, Departement of Gastroenterology, Mahmoud Matri Hospital, Ariana, 2080, Tunisia; 2 University of Tunis El Manar, Departement of Bacteriology & Biochemistry Laboratory, Mahmoud Matri Hospital, Ariana, 2080, Tunisia; 3 University of Tunis El Manar, Departement of Anatomopathology, Abderrahmen Mami Hospital, Ariana, 2080, Tunisia; 4 Clinical Analysis Laboratory “Fendri”, Ariana, 2080, Tunisia; 5 University of Tunis El Manar, Dacima Consulting, Tunis, 2080, Tunisia

**Keywords:** eradication, *Helicobacter pylori*, quadruple therapy

## Abstract

The treatment of *Helicobacter pylori* infection remains a challenge. None of the proposed treatment regimens has resulted in a 100% eradication rate. The aim of our study was to compare the rate of *H. pylori* eradication after standard or dose-optimized amoxicillin quadruple therapy. We conducted a prospective comparative study collating patients naive to any anti-*H. pylori* treatment and with chronic *H. pylori* infection documented by histological examination. Patients were randomly assigned to either standard quadruple therapy or optimized quadruple therapy. Eradication control was performed by urea breath test. Eighty-eight eligible patients were included with 44 in each group.There was no significant difference between the eradication rates of Qo-14 and Qs-14 neither in ITT (84 vs 70.4%; p = 0.127) nor in PP (82.1 vs 77.7%; p = 0.473). Compliance and tolerance appeared similar in each group.

## Plain language summary

*H. pylori* is a common bacterium that can cause several digestive infections, including gastric ulcers and gastric cancer. The aim of this study was to compare the rate of *H. pylori* eradication after a standard dose compared with a double dose of a specific therapy known as amoxicillin quadruple therapy. The results showed no significant difference between the eradication rates of standard or optimized quadruple therapy.

## Abstract (plain language summary)

There was no significant difference between the eradication rates of Qo-14 and Qs-14 neither in intention-to-treat nor in per protocol.

Compliance and tolerance appeared similar in each group.

Poor socioeconomic conditions, as well as rural origin were the two predictive factors of *H. pylori* eradication failure.

Chronic gastric *Helicobacter pylori* (*H. pylori*) infection is one of the most widespread bacterial infections globally, affecting nearly half of the world's population [1,2]. Its prevalence appears to be closely linked to the living standards of different countries and their socio-economic conditions [3]. In Tunisia, its prevalence has been estimated at 64% among blood donors [4] and at 51.4% among first-year primary school children [5].

*H. pylori* infection is implicated in various digestive disorders, including gastric and duodenal ulcers, non-ulcer dyspepsia, gastric cancer and mucosa-associated lymphoid tissue (MALT) lymphoma [6,7]. Treatment involves a combination of therapies targeting stomach acid production and antibiotic treatment. However, the limited effectiveness of antibiotics against *H. pylori*, combined with a global rise in resistance, particularly to clarithromycin, has led to diverse therapeutic approaches that vary from one country to another, depending on their own bacterial ecology.

In Tunisia, the most common regimen adopted in first-line treatment is non-bismuth quadruple therapy, based on amoxicillin, metronidazole and clarithromycin, in combination with PPI (proton pump inhibitor). In fact, among the antibiotics active on *H. pylori*, amoxicillin occupies a major place due to its minimal resistance; however, it is typically administered at a rate of one gram every 12 h, while optimal pharmacokinetic efficiency often recommends administration every 6–8 h. Recent suggestions propose a daily dose of 3 gm of amoxicillin for improved eradication rates. Recently, authors have suggested the use of a dose of 3 g per day of amoxicillin for a better eradication rate [8]. The current study aims to compare the *H. pylori* eradication rate following standard concomitant quadruple therapy with 2 g of amoxicillin versus optimized quadruple therapy with 3 g of amoxicillin in Tunisian population and identify different factors associated with a poor therapeutic response.

## Methods

### Subjects & study design

This study was a prospective, randomized, single-center investigation conducted at the hepato-gastroenterology department of Mahmoud Matri Hospital in Ariana, in collaboration with the bacteriology and biochemistry laboratory of the same hospital, the anatomopathology department of Abderrahmen Mami Hospital in Ariana, and the private clinical analysis laboratory ‘Fendri’. The study was conducted from January 2019 to December 2019 and included all patients aged 18 to 65 years with documented *H. pylori* infection confirmed through pathological examination of per-endoscopic gastric biopsies. Our study focused on individuals who had undergone upper endoscopy (UE) and for whom the search for *H. pylori* was indicated according to the European Maastricht IV recommendations [9].

Written informed consent was obtained from all participating subjects, and the study received approval from the local ethics committee. The research adhered to the recommendations of the CONSORT statement for ensuring the quality of reporting randomized control trials. The study was conducted using the Dacima software.

Non-inclusion criteria were:

Patients who had:

▪ Previously received *H. pylori* eradication therapy;
▪ Known allergy to one of the components of the kit used (Helikit) for the 13C-urea breath test (UBT);
▪ A contraindication to 13C-UBT;
▪ Allergy or contraindications to the following antibiotics: amoxicillin, clarithromycin, metronidazole and quinolones;
▪ Received antibiotics during the 4 weeks, or PPI during the 2 weeks prior to inclusion in the study;
▪ A history of bariatric surgery;
▪ Active gastrointestinal bleeding;
▪ Severe comorbidities such as decompensated cirrhosis, end-stage renal disease, decompensated cardiac disease and neoplastic disease;
▪ Patients on long-term immunosuppressive or corticosteroid therapy at a dose greater than 20 mg/day;
▪ Pregnant or lactating women.

Were excluded from this study:

▪ Patients with poor compliance;
▪ Patients refusing to perform the control by 13C-UBT.

### Data collection

For each patient, the following data were collected: demographic and clinical data including medical personal history, family history of digestive neoplasia, tobacco and alcohol consumption, use of gastro-aggressive drugs (NSAIDs and/or low-dose aspirin) and indication for upper endoscopy (Appendix 1).

Another questionnaire (Appendix 2) was used to record therapeutic compliance (good, bad) as well as occurrence and type of adverse events.

### Randomization & therapy regimen

Eligible subjects were randomly assigned in a 1:1 ratio to receive either standard quadruple therapy (Qs-14) including: amoxicillin 1 g twice daily, clarithromycin 500 mg twice daily, metronidazole 500 mg twice daily and esomeprazole 40 mg twice daily for 14 days, or optimized quadruple therapy (Qo-14) including: amoxicillin 1 g three-times daily, clarithromycin 500 mg twice daily, metronidazole 500 mg twice daily and esomeprazole 40 mg twice daily for 14 days. Eradication control was performed by 13C-UBT at least 4 weeks after the end of the treatment.

Esomeprazole was given 30 min before meals, and other antibiotics were given during meals. Side effects and compliance were evaluated through a questionnaire administered upon completion of the therapy (questionnaire no. 1). Compliance was defined good when more than 90% of the total pills were taken. Side effects were categorized based on their impact on daily life, with classifications including ‘mild’ (transient and well-tolerated), ‘moderate’ (resulting in discomfort that partially interfered with daily life), or ‘severe’ (causing substantial interference with daily life).

### Statistical analysis

Data were entered and analyzed using SPSS (Statistical Package for the Social Sciences) version 22 software. Continuous variables were described by mean and standard deviation, and qualitative data were described by percentages. Cure rates were evaluated by intention-to-treat (ITT): consists of analyzing all patients in the group in which they were randomized, and per-protocol (PP) analysis: consists of analyzing a subgroup of the population including only patients in perfect compliance with the protocol. Differences between groups were evaluated by Student's test for continuous variables and Chi-square test or Fisher's exact test for categorical variables. p < 0.05 was considered statistically significant.

To determine the associated factors affecting the treatment response, clinical, endoscopic, histological and bacterial factors were analyzed by univariate analysis. These variables included the following: age (< = 50 years), female gender, BMI > = 25 kg/m^2^, presence of diabetes, history of smoking, history of alcohol consumption, low level of education, poor socio-economic conditions, history of peptic ulcer disease, high density of *H. pylori* and rural origin. The variables found to be significant by univariate analysis were subsequently assessed by a stepwise logistic regression method to identify independent factors for eradication outcome.

### Outcomes

The primary outcome of this study was the eradication rate in each group. At least 4 weeks after completion of treatment, *H. pylori* status was determined by 13C-UBT. Eradication was defined as negative from 13C-UBT. Secondary outcomes were the prevalence of adverse events, compliance, and related factors (such as gender, age, body mass index, smoking, low level of education, poor socio-economic conditions, peptic ulcer disease or high density of *H. pylori* and rural origin) that could potentially influence *H. pylori* eradication rate.

## Results

### Characteristics of the study groups

**Figure 1.**
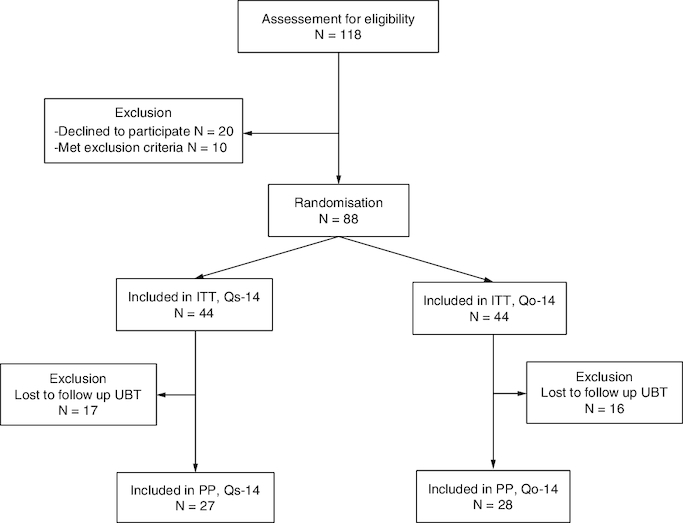
Flow diagram of this study. ITT: Intention-to-treat; PP: Per-protocol; Qs-14: Standard quadruple therapy including 2 g amoxcillin; Qo-14: Optimized quadruple therapy including 3 g amoxcillin; UBT: 13C-urea breath test.

As shown in Figure 1, a total of 118 eligible *H. pylori*-infected patients were invited; 88 patients were enrolled (N = 44 per group) in the ITT analysis. Ultimately, 17 patients were lost during follow-up in the Qs-14 group and 16 patients in the Qo-14 group, resulting in 27 in the Qs-14 group and 28 in the Qo-14 group. The demographic data of the two groups were similar (Table 1).

**Table 1.** Demographic data and endoscopic appearance of the two groups of patients.

Characteristics	Qs-14 (n = 44)	Qo-14 (n = 44)	p-value
Age (year) (mean+/- SD)	39.5 ± 12.38	44 ± 12.50	0.443
Gender (male/female)	16/28	17/27	0.826
Comorbidity	19 (43.2%)	21 (47.7%)	0.669
**Endoscopic findings**			
Non-nodular congestive gastropathy	34 (77.2%)	36 (81.8%)	0.597
Congestive and nodular gastropathy	8 (18.1%)	7 (15.9%)	0.777
Erosive gastritis	2 (4.5%)	2 (4.5%)	0.692
Bulbar ulcer	5 (11.3%)	7 (15.9%)	0.534
Gastric ulcer	0 (0%)	1 (2.3%)	0.500

### Eradication of *H. pylori*

Among the 44 patients initially included in the Qs-14 group, complete resolution of symptoms was noted in 37 patients (84%); as for the 44 patients included in the Qo-14 group, 31 patients (70,4%) have completely improved their symptoms.

Considering the 27 patients in the Qs-14 group who underwent an eradication control through an UBT, *H. pylori* eradication was achieved in 21 of them, representing a rate of 77.78%.

As for the 28 patients in the Qo-14 group who underwent an eradication control through an UBT, *H. pylori* eradication was achieved in 23 of them, representing a rate of 82,1%.

Table 2 lists the eradication rates of the Qs-14 and Qo-14 groups. In ITT, the *H. pylori* eradication rate was 84% (37/44) in the Qo-14 group and 70.4% (31/44) in the Qs-14 group (p = 0.127). In PP, it was 82.1% (23/28) and 77.7% (21/27) in the Qo-14 and Qs-14 groups, respectively (p = 0.473), without a statistically significant difference.

**Table 2.** The major outcomes of the two groups of patients.

Characteristics	Qs-14 (n = 44)	Eradication rate Qo-14 (n = 44)	p-value
ITT	31/44 (70.4%)	37/44 (84%)	0.127
PP	21/27 (77.7%)	23/28 (82.1%)	0.473
Adverse events	8/44 (18.2%)	3/44 (6.8%)	0.5

### Adverse events & factors influencing the efficacy of *H. pylori* eradication therapy

In our study, only 12% of the patients had developed adverse events following anti-*H. pylori* quadruple therapy, affecting more women than men, with a rate of 64% versus 36%.The adverse event rates were 18.2% in the Qs-14 group and 6.8% in the Qo-14 group (p = 0.5) (Table 2). These adverse events included dysgeusia, dyspepsia, nausea, vomiting, diarrhea, asthenia, facial edema and headache (Table 3); however, these were mild and did not markedly disturb the patients' daily activities. Therefore, both groups had good drug compliance (100%). As for factors influencing the efficacy of *H. pylori* treatment, failure of *H. pylori* eradication was observed in 11 patients (20%) of the study population who could benefit from a control 13C-UBT. Univariate analysis showed that poor socioeconomic conditions, as well as rural origin were found to be the two predictive factors of *H. pylori* eradication failure in our study population (p = 0.002) and (p = 0.008), respectively, as shown in Table 4. Logistic regression showed that poor socioeconomic conditions were the only independent predictor factor of treatment failure (OR: 1.09; 95% CI: [0.417–2.873]).

**Table 3.** Adverse events in the two groups of patients.

Adverse events (AE)	Number of AE (%) Qs-14 (n = 44)	Number of AE (%) Qo-14 (n = 44)	p-value
Dysgeusia	1 (2.3%)	0 (0%)	0.500
Dyspepsia	0 (0%)	1 (2.3%)	0.500
Nausea	1 (2.3%)	1 (2.3%)	0.500
Vomiting	1 (2.3%)	0 (0%)	0.534
Diarrhea	2 (4.5%)	1 (2.3%)	0.500
Asthenia	1 (2.3%)	0 (0%)	0.500
Facial edema	1 (2.3%)	0 (0%)	0.500
Headache	1 (2,3%)	0 (0%)	0.500

**Table 4.** Factors influencing the efficacy of *H. pylori* eradication.

Principal parameters	Study population: n = 55		
	Eradication failure	Eradication success	p-value
Age = <50 years	7/11 (63.6%)	27/44 (61.3%)	0.770
Female gender	7/11 (63.6%)	26/44 (59%)	0.783
BMI >=25 kg/m^2^	7/11 (63.6%)	28/44 (63.6%)	0.518
Diabetes	0/11 (0%)	6/44 (13.6%)	0.244
Tobocco	3/11 (27.2%)	10/44 (22.7%)	0.751
Alcohol	1/11 (9%)	5/44 (11.3%)	0.656
Low level of education: illiterate/primary	6/11 (54.5%)	17/44 (38.6%)	0.2
Poor socio-economic conditions	11/11 (100%)	31/44 (70.4%)	**0.002** ^†^
Peptic ulcer disease	1/11 (9%)	6/44 (13.6%)	0.686
High density of *H. pylori*	2/11 (18.1%)	3/44 (6.8%)	0.241
Rural origin	3/11 (27.2%)	1/44 (2.27%)	**0.008** ^†^

† Bold values indicate the statically significant values.

## Discussion

It has recently been shown that the most effective approach to enhance the eradication rate of *H. pylori* is a personalized treatment based on antibiotic susceptibility [10]. However, this strategy may not be feasible in many developing countries. Therefore, many studies have focused on optimizing the recommended treatment regimens.

The fundamental concept behind optimized quadruple therapy is to create ideal conditions for *H. pylori* eradication. This involves ensuring that antibacterial agents reach the infection site in an active form and at a bactericidal concentration [11].

Assuming that resistance to amoxicillin, whether primary or acquired, is exceptional [12,13], increasing the dose of amoxicillin helps overcome the problem of bioavailability at the site of infection. Indeed, *H. pylori* occupies a protected ecological niche, since it colonizes the superficial layers of the gastric epithelium as well as the deep layers of the mucus [11].

Despite the common recommendation of using amoxicillin at a dose of 1 g twice daily in all treatment regimens, pharmacokinetic data support the use of higher doses and more frequent administration. After oral administration, the local half-life of amoxicillin in the stomach is brief, and most of its action occurs through plasma transfer. A Chinese study published in 2010, showed that the peak concentrations of amoxicillin in gastric juice and mucosa were significantly lower than those in plasma, supporting the need for higher doses for *H. pylori* eradication [14]. Similarly, administering 1 g of amoxicillin three times a day improved treatment success, as a single dose did not maintain bactericidal concentration in the mucus for more than one hour [8].

The results of our study showed that there was no significant difference between the eradication rates of Qo-14 and Qs-14, neither on ITT, nor on PP, although there is a trend toward the superiority of optimized doses. This needs to be confirmed by a larger sample size study, especially as some recent recommendations are in favor of adapting amoxicillin doses to weight (50 mg/kg/d) in three or even four doses.

By reviewing the literature, most of studies investigating this optimization strategy in terms of amoxicillin dose have shown successful results.

In 2016, Zullo *et al.* [15] included in their study consecutive naive *H. pylori*-infected patients, who underwent an upper endoscopy in four Italian hospitals due to dyspeptic symptoms and found to be infected at routine histological assessment. Patients enrolled received a 10-day, high-dose dual therapy comprising esomeprazole (40 mg t.i.d) and amoxicillin (1 g t.i.d.). The overall eradication was 87.5% without a statistically significant difference among centers.

Similarly, a Chinese study, published in 2020, which included 216 patients receiving 14-day triple therapy containing amoxicillin 1 g and metronidazole 400 mg taken three times daily with esomeprazole 20 mg twice daily, with or without bismuth 220 mg twice daily, showed an eradication rate of *H. pylori* in PP with bismuth of 97.9% (94/96; 95% CI: 95.1–100%) and without bismuth of 94.7% (90/95; 95% CI: 90.3–99.1%) (p = 0.43); such a high eradication rate, especially without bismuth, could precisely be attributed to the use of high-dose amoxicillin [16].

A meta-analysis including ninety-four studies (8061 patients) comparing PPI-amoxicillin-metronidazole (PAM) regimens with PPI-amoxicillin-clarithromycin regimens indicated that the results of PAM triple regimens containing low-dose amoxicillin were inferior to those of PAM triple regimens containing high-dose amoxicillin (75% vs 81%), but with the same dose of metronidazole, suggesting that amoxicillin played an important role in treatment efficacy [17].

To our knowledge, although most of the above-mentioned studies argue in favor of increasing amoxicillin doses in the treatment of *H. pylori* eradication, no study to date has made a direct comparison between standard concomitant quadruple therapy and optimized quadruple therapy in terms of amoxicillin doses, as in our study.

As for tolerance, in our study, only 12% of the patients had developed adverse events following anti-*H. pylori* quadruple therapy, with no significant difference between the two groups (6.8% versus 18.2% in the Qo-14 and Qs-14 groups, respectively [p = 0.107]). Similarly, no cases of treatment discontinuation related to these adverse effects were noted among our patients.

A Chinese meta-analysis published in 2016, studying the efficacy and tolerance of concomitant quadruple therapy retained a higher incidence of occurrence of side effects, compared with hybrid quadruple therapy (44.2% vs 39%) [18]. This could be explained by the shorter duration of use of clarithromycin and metronidazole in hybrid therapy. As for amoxicillin, it was prescribed for the same duration in both courses, which is an additional argument for the safety of this molecule.

Similarly, an Italian meta-analysis published in 2015, which investigated the efficacy of dual therapy involving a daily dose of 3 g of amoxicillin, revealed that adverse events associated with this treatment approach were observed in 8.9%. This finding aligns with the results of the current study [15].

Thus, increasing the dose of amoxicillin does not seem to be associated with a higher incidence of adverse events.

In terms of factors influencing the eradication rate, our study identified poor socioeconomic conditions and rural origin as two predictive factors for *H. pylori* eradication failure in our study population. Indeed, the literature supports the consideration of low socioeconomic status as a factor associated with eradication treatment failure. For instance, Itskoviz *et al.* demonstrated a significant association between poor socioeconomic conditions and *H. pylori* eradication treatment failure (OR: 1.24; 95% CI: 1.17–1.31, p < 0.001) [19]. Similarly, a Chinese study published in 2020, showed that better income status was a factor in good response to treatment. By reviewing the literature, the association between rural origin and a lower eradication rate of *H. pylori* was also evident. A 2021 Spanish study, involving 693 patients, revealed that individuals residing in rural areas faced a higher risk of *H. pylori* eradication failure compared with urban residents (OR: 1.59; 95% CI: 1.05–2.41) [20].

### Strengths & limitations of the study

To the best of our knowledge, this is the first prospective randomized study conducted in Tunisia and globally, comparing optimized quadruple therapy in terms of amoxicillin doses versus concomitant quadruple therapy.

Our study has nevertheless some limitations. Firstly, we did not employ histological studies for the verification of *H. pylori* eradication, preventing the assessment of the progression of chronic gastritis lesions and pre-cancerous lesions following eradication treatment. Additionally, this was a single-center study with a relatively small number of patients, emphasizing the importance of further research to validate and extend our findings.

## Conclusion

In conclusion, this study shows a comparable eradication rate of *H. pylori* after standard or optimized quadruple therapy, with nevertheless, a trend in favor of the optimized treatment without additional adverse effects. As for factors influencing eradication rate, our study found that poor socioeconomic conditions, as well as rural origin were the two predictive factors of *H. pylori* eradication failure in our study population. Larger studies evaluating the efficacy of optimized quadruple therapy regimen are warranted, notably in countries where bismuth quadruple therapy is not always available.
